# Flavonoid-Rich Orange Juice Intake and Altered Gut Microbiome in Young Adults with Depressive Symptom: A Randomized Controlled Study

**DOI:** 10.3390/nu12061815

**Published:** 2020-06-18

**Authors:** Miey Park, Jihee Choi, Hae-Jeung Lee

**Affiliations:** 1 Department of Food and Nutrition, College of BioNano Technology, Gachon University, Gyeonggi-do 13120, Korea; mieyp@naver.com (M.P.); mongtaengi@naver.com (J.C.); 2 Institute for Aging and Clinical Nutrition Research, Gachon University, Gyeonggi-do 13120, Korea

**Keywords:** depression, microbiota, flavonoid treatment, Lachnospiraceae

## Abstract

Depression is not just a general mental health problem but a serious medical illness that can worsen without treatment. The gut microbiome plays a major role in the two-way communication system between the intestines and brain. The current study examined the effects of flavonoids on depression by observing the changes in the gut microbiome and depressive symptoms of young participants consuming flavonoid-rich orange juice. The depressive symptom was assessed using the Center for Epidemiological Studies Depression Scale (CES-D), a psychiatric screening tool used to detect preexisting mental disorders. The study population was randomly divided into two groups: the flavonoid-rich orange juice (FR) and an equicaloric flavonoid-low orange cordial (FL) group. For 8 weeks, participants consumed FR (serving a daily 380 mL, 600 ± 5.4 mg flavonoids) or FL (serving a daily 380 mL, 108 ± 2.6 mg flavonoids). In total, 80 fecal samples from 40 participants (mean age, 21.83 years) were sequenced. Regarding depression, we observed positive correlations between brain-derived neurotrophic factor (BDNF) and the Lachnospiraceae family (*Lachnospiraceae_uc* and *Murimonas*) before flavonoid orange juice treatment. Most notably, the abundance of the Lachnospiraceae family (*Lachnospiraceae_uc*, *Eubacterium_g4*, *Roseburia_uc*, *Coprococcus_g2_uc*, *Agathobacter_uc*) increased after FR treatment compared to that after FL treatment. We also validated the presence of unclassified Lachnospiraceae through sensitive real-time quantitative polymerase chain reaction using stool samples from participants before and after flavonoid treatment. Our results provide novel interventional evidence that alteration in the microbiome due to flavonoid treatment is related to a potential improvement in depression in young adults.

## 1. Introduction

Depression is a common psychiatric disorder and is accompanied by symptoms such as sadness, decrease in motivation or interest, low self-esteem, sleep deprivation, loss of appetite, fatigue, and loss of concentration [1]. Moreover, depression can lead to serious problems in daily or social life and may also result in suicide. It is being increasingly recognized that few clinical symptoms are found in healthy populations [2]. It is estimated that the number of individuals with depression worldwide is 350 million, and the population affected by depression is gradually expanding [1,3]. 

Dietary patterns and depression are closely related [4,5]. Previous studies have reported that the intake of sweets, red and processed meat, high-fat dairy products, and refined grains, and low intake of fruits and vegetables may increase the risk of depression [5,6,7]. In particular, flavonoids and polyphenols (micronutrients) are abundant in fruits, vegetables, tea, and cocoa. Previous studies have revealed that flavonoid intake increases the amount of blood delivered to the brain, thereby improving brain cognitive function [8,9,10,11]. Epidemiological studies [12,13] and clinical studies [14,15] have also reported that high flavonoid intake through vegetables and fruits helps lower the risk of depression. Although fruits and fruit juices can easily supply flavonoids, such as hesperidin and narirutin, studies on their effectiveness on the human body are still at an early stage, and further studies are needed.

There have been an increasing number of observational studies on the associations between habitual quality of diet and the prevalence of depression [16]. Additionally, it has been reported that dietary flavonoids (polyphenols) are associated with gut microbiome regulation, and these microbiomes can prevent and treat depression by increasing the synthesis of serotonin, which is a neurotransmitter in the body [16,17,18]. An experimental animal model showed that probiotics may reduce pro-inflammatory immune responses and increase levels of the serotonergic precursor tryptophan, thereby ameliorating depressive symptoms [19]. Furthermore, depression was found to be reversed by administering the probiotic *Bifidobacterium infantis* to male Sprague–Dawley rats (*n* = 20) [20]. 

Tryptophan, as a precursor, is an essential amino acid needed by the body to synthesize serotonin [18]. As an essential amino acid, tryptophan is not synthesized from simple substances in humans and other animals; thus, it must be obtained through protein-based foods and dietary proteins. Microbes and plants generally synthesize tryptophan from shikimic acid or anthranilate, and human gastrointestinal microbiota metabolize tryptophan into indole and subsequently 3-indolepropionic acid, a powerful neuroprotective antioxidant that scavenges free radicals [21,22,23].

Probiotics, including *Bifidobacteria* and *Lactobacilli*, also alleviate the immune response, decrease pathogens, and maintain the intestinal microbiota in subjects receiving antibiotic treatment [24,25,26]. It plays a major role in two-way communication between the intestines and brain, named the gut–brain axis [26,27]. Moreover, the impact of probiotics on human psychiatric disorders has recently emerged as an area of interest in neuroscience [28]. 

The activity of microbial metabolites is mainly due to their ability to permeate the blood–brain barrier. Dietary polyphenols are metabolized by microorganisms in the colon, and microbial metabolites of polyphenol, isoflavones, and lignans generally show greater permeability through the man-made gut and blood–brain barrier than their parent compound [29]. Another polyphenol metabolite, gallic acid derivatives, demonstrated neuroprotective effects through the modulation of the nuclear factor κB (NF-κB) pathway [30]. Polyphenols may affect the composition of the gut microbiota, and bioactive compounds in polyphenol metabolites produce clinical benefits [31].

Although previous studies have indicated that flavonoids consumed through the diet could affect gut microbiome regulation and depression, there have been limited intervention studies on the microbial metabolites of polyphenol. Therefore, this study aimed to evaluate the effects of flavonoids on depression in young adults by observing the changes in the gut microbiome and depressive symptoms of participants consuming flavonoid-rich orange juice. Furthermore, this study attempted to establish a theoretical basis for providing basic data and policy direction to develop a dietary program for alleviating and preventing depressive symptoms.

## 2. Materials and Methods

### 2.1. Participants

Forty participants (age: 20–30 years; mean age: 21.83 years with a SD of 2.43) were recruited from the population living in Seoul and Gyeonggi-do from April to June 2018. The power calculation indicated that the minimum number of participants for each group was 17. This study recruited 40 participants for 2 groups (20 participants each) by considering the dropout rate of 20%. The final 40 participants included 16 men and 24 women (Table S1). Depression symptoms were assessed using the Center for Epidemiological Studies Depression Scale (CES-D), which is a psychiatric screening tool to detect preexisting psychiatric disorders and has been used extensively in population-based studies [32,33,34]. All participants with CES-D scores ≥21 were indicated as depression in our study [35]. All participants provided consent for participation after understanding the objective of this study, test schedule, and potential risk. Those who were using antibiotics, had a family history of mental illness, had been diagnosed with psychiatric disorders, had a history of antipsychotic medication use, or had a bowel disease were excluded from the study. All procedures were conducted in accordance with the Declaration of Helsinki [36] and approved by the Gachon University Institutional Review Board (IRB, 1044396-201803-HR-074-01).

### 2.2. Intervention Study Design

The study had a randomized, single-blind design and was conducted for 8 weeks. The volunteers were randomly divided into 2 groups of 20 participants each, the FR and FL groups. The FR group consumed flavonoid-rich orange fresh juice (190 mL each) and the FL group consumed orange flavored cordial drink (190 mL each) twice daily (30–60 min before breakfast and dinner) for 8 weeks. Daily orange juice intake was monitored by phone and short message service (SMS), one to one by researchers. The participants visited the Aging and Clinical Nutrition Research Institute in Gyeonggi-do for screening 1 week before the beginning of the treatment and underwent the CES-D test. To check whether they met the inclusion and exclusion criteria, the registered participants returned on the first day of the treatment (1 week after the screening visit) and provided fecal specimens in a fecal collection bag or accessory bag. The 24 h dietary recall evaluation (Table S2); food frequency questionnaire (FFQ) [37]; and 24 h dietary recall evaluation, blood tests, and anthropometric assessments using InBody 720 (Biospace Co. Ltd., Seoul, Korea) were performed (Table S2). The participants also underwent the same tests at the end of the treatment (after 8 weeks).

### 2.3. Treatment Drinks

This study used a commercially available, 100% pure, Florida orange juice (Natalie’s Orchid Island Juice Co. and Tomato Agricultural Association Corporation Inc., Korea) as the FR drink (190 mL) and a commercially available orange-flavored cordial (Del Monte Foods Inc. and Lotte Chilsung Beverage Co. Ltd., Seoul, Korea) as the FL drink (190 mL). The total flavonoid content was analyzed by high-performance liquid chromatography–mass spectrometry. The total flavonoid content in the orange drink was 157.9 ± 1.4 mg/100 g and that in the flavored drink was 28.4 ± 0.7 mg/100 g (Table S3). The two drinks were almost identical in appearance, volume, taste, and calories, as well as in glucose, fructose, and sucrose levels, except in the flavonoid content (Table S4).

### 2.4. Blood Tests

Participants were asked during screening to avoid certain foods (e.g., high-fat and high-sucrose foods, berries, fruits, fruit juice, tea, jams, and alcohol) and to have an low-fat diet for 24 h before the blood test. Fasting blood samples (5 mL of blood) were collected at the beginning (baseline) and at the end (8 weeks) of the treatment. Serum obtained from each blood sample was divided into 500 μL aliquots and was used to measure brain-derived neurotrophic factor (BDNF), serotonin, folate, homocysteine, high-sensitivity C-reactive protein (hs-CRP), and vitamin B12 levels (Table 1). All blood parameters were analyzed by SQLab (SQLab Co., Giheung-gu, Korea).

### 2.5. Dietary Intake

Twenty-four hour dietary recall and FFQ were measured twice by a qualified dietician. Dietary intake assessment was conducted by 24 h recall method and nutrient intakes were analyzed by the Computer-Aided Nutritional analysis program for professionals (CAN-Pro 4.0 program; Korean Nutrition Society, Seoul, Korea). FFQ was conducted to check the dietary pattern during the intervention. No separate major dietary intervention or physical activity program was offered. Moreover, participants were advised to maintain physical activities during the trial.

### 2.6. Fecal Sample Collection and DNA Extraction

The participants provided fecal specimens at the beginning (before flavonoid treatment group; depression, *n* = 40) and at the end (after flavonoid treatment groups; FR, *n* = 20 and FL, *n* = 20) of the 8-week treatment. Fecal samples were collected using the fecal collection kit. DNA from the fecal samples was extracted using a Fast DNA SPIN extraction kit (MP Biomedicals, Solon, Ohio, USA) according to the manufacturer’s instructions. The PCR amplification and sequencing methods used in this study have been previously described [38]. Briefly, the V3 and V4 regions of the 16S ribosomal RNA gene were amplified and sequenced using Illumina MiSeq Sequencing System (Illumina, SD, USA) by ChunLab, Inc. (Seoul, Korea).

### 2.7. PCR Amplification and Illumina Sequencing

The first PCR amplification was performed using a T100 thermal cycler (Bio-Rad, Hercules, CA, USA) to amplify the V3 and V4 regions of the 16S rRNA gene. The primers used were 341F (5′-TCGTCGGCAGCGTC-AGATGTGTATAAGAGACAG-CCTACGGGNGGCWGCAG-3′; the underlined sequence indicates the target region primer) and 805R (5′-GTCTCGTGG GCTCGG-AGATGTGTATAAGAGACAG-GACTACHVGGGTATCTAATCC-3′). The first PCR amplification was conducted under the following conditions: initial denaturation at 95 °C for 3 min followed by 25 cycles of denaturation at 95 °C for 30 s, primer annealing at 55 °C for 30 s, and extension at 72 °C for 30 s, with a final elongation at 72 °C for 5 min. The second PCR amplification to attach the Illumina NexTera barcodes was performed with the i5 forward primer (5′-AATGATACGGCGACCACCGAGATCTACAC-XXXXXXXX-TCGT CGGCAGCGTC-3′; X indicates the barcode region) and i7 reverse primer (5′-CAAGCAGAAGACGGCATACGAGAT-XXXXXXXX-AGTCTCGTGGGCTCxGG-3′). Conditions used for the second amplification reaction were the same as those described for the first reaction, except only 8 amplification cycles were performed. The PCR product was confirmed using 1% agarose gel electrophoresis and visualized under a Gel Doc system (BioRad, Hercules, CA, USA). The amplified products were purified using the QIAquick PCR Purification Kit (Qiagen, Valencia, CA, USA). Equal concentrations of purified products were pooled together, and short fragments (non-target products) were removed using the Ampure beads kit (Agencourt Bioscience, MA, USA). The quality and product size were assessed on a Bioanalyzer 2100 (Agilent, Palo Alto, CA, USA) using a DNA 7500 chip. Mixed amplicons were pooled, and sequencing was performed by ChunLab, Inc. (Seoul, Korea), using the Illumina MiSeq Sequencing System (Illumina, SD, USA) according to the manufacturer’s instructions.

### 2.8. Classification of Microbiome

Raw reads were checked for quality, and low-quality reads (<Q25) were filtered using Trimmomatic 0.32 [39]. After the quality control process, paired-end sequence data were merged together using PANDAseq [40]. Then, primers were trimmed using a proprietary program of ChunLab at a similarity cutoff of 0.8. Nonspecific amplicons that did not encode 16S rRNA were detected by HMMER’s hmmsearch program with 16S rRNA profiles [41]. Sequences were denoised using DUDE-Seq, and non-redundant reads were extracted by UCLUST clustering [42,43]. The EzBioCloud database was used for taxonomic assignment using USEARCH (8.1.1861_i86linux32) followed by more precise pairwise alignment [43,44]. UCHIME [45] and the nonchimeric 16S rRNA database from EzBioCloud were used to detect chimeras on reads with a best hit similarity rate <97%. Sequence data were then clustered using CD-HIT [46] and UCLUST [43]. The alpha- and beta-diversity analyses with the Chao, Phylogenetic Diversity, Shannon, and Simpson indexes were conducted using BIOiPLUG, which is ChunLab’s bioinformatics cloud platform.

### 2.9. Preparation of Genomic DNA from Reference Strains and Fecal Samples

Fecal microbial DNA from 200 mg fecal samples was prepared using the QIAamp DNA Stool Mini Kit (Qiagen, Hilden, Germany) according to the manufacturer’s instructions. DNA was quantified using a NanoDrop ND-1000 spectrophotometer (Thermo Electron) and stored at −20 °C before analysis.

### 2.10. Real-Time Quantitative PCR

Real-time quantitative PCR was conducted using a LightCycler 480 (Roche, Germany), and the group and species-specific primers for PCR are presented in Table S5 [47]. The primers were verified using Primer3, and were synthesized commercially. Quantitative PCR was performed in 96-well plates with final volumes of 20 μL, consisting of 1 μL of fecal DNA, 0.5 μL of primers (10 pmol each), 10 μL SYBR Green I master (Roche, Mannheim, Germany), and 8 μL of H_2_O. PCR amplification involved pre-incubation at 94 °C for 4 min, followed by 55 cycles of amplification (denaturation at 94 °C for 15 s, primer annealing at 55 °C for 15 s, and elongation at 72 °C for 20 s). Melting curves were obtained by heating samples from 50 to 90 °C at a rate of 5 °C/s.

### 2.11. Statistical Analysis

Differences in dietary intake before and after 8 week-intervention were determined using the paired t-test. Between groups with changes before and after treatment, the two sample t-test or Wilcoxon signed-rank test was used by SPSS (ver. 23.0; SPSS Inc., IL, USA). Results are expressed as mean ± SEM, and statistical significance was determined at *p* < 0.05 and *p* < 0.01, respectively.

To investigate the association of BDNF levels and clinical index of depression symptoms with Gut Microbiota, Spearman’s rank correlation analysis was used to calculate the correlation coefficient (r) between the intestinal microflora and biomarkers. The heat map was plotted in 35 genera for participants with BDNF levels and statistically significant results (*p* < 0.1). All statistical analyses were performed using SAS (ver. 23.0; SAS Inc., IL, USA). 

## 3. Results

### 3.1. Characteristics of the Study Participants

The study participants (*n* = 40) consisted of 16 men (40%) and 24 women (60%). The mean age was 21.83 ± 2.43 years, and there was no dropout. Statistical analysis was conducted for participant characteristics. Baseline characteristics did not differ between participants in the FR (*n* = 20) and FL (*n* = 20) groups (Table S1).

### 3.2. Nutrient Intakes of 24 h Recall

The results of the one-day 24 h recall showed that in the FR group, only the intake of energy, protein, fat, phosphate, sodium, and vitamin B12 decreased significantly (*p* < 0.05), and that of folate and vitamin C increased significantly (*p* < 0.05). In the FL group, the intake of riboflavin and niacin significantly decreased (*p* < 0.05), while that of other nutrients did not change. The intake of folate and vitamin C intake was significantly (*p* < 0.05) different between the FR and FL groups (Table S2).

### 3.3. Comparison of Hematological Profiles and Anthropometric Measurements

Blood test results revealed an increase in brain-derived neurotrophic factor (BDNF) levels in the FR group (Table 1). The numerical increase in the levels in the FR group was larger than that in the FL group, but was not significantly different (*p* < 0.05). The serotonin level in the FR group was high but was not significantly higher than that in the FL group, and there was no significant difference between the two groups (*p* = 0.058). Folate and homocysteine levels showed significant differences at baseline and post-intervention in the FR group (*p* < 0.05). Homocysteine level in the FL group was significantly different and fell out of the normal range (5.0–15.0 μmol/L). Moreover, after adjusting for sex, age, and household income, body fat (%), homocysteine level, and high-sensitivity C-reactive protein (hs-CRP) level were significantly different (*p* < 0.05) between the FR and FL groups (Table 1).

### 3.4. Comparison of the Center for Epidemiological Studies Depression Scale Scores

Post-intervention, the mean Center for Epidemiological Studies Depression Scale (CES-D) scores in the FR and FL groups decreased to <20 points (Table 1). Moreover, adjusted (sex, age, education, and household income) multiple regression analysis showed that the *p*-values of the CES-D score in the FR group decreased significantly (*p* < 0.0001) compared to that of FL groups (*p* < 0.001) after the intervention.

### 3.5. Sequencing Characteristics and Changes in Microbial Diversity in Depression Symptoms Group

In total, 80 fecal samples from 40 participants before and after the intervention were sequenced on the Illumina MiSeq sequencer. The sequencing results were obtained from 40 untreated participants (CES-D score ≥ 21), who were randomly divided into the FR group (20 participants) and FR group (20 participants) for the analysis.

**Figure 1:**
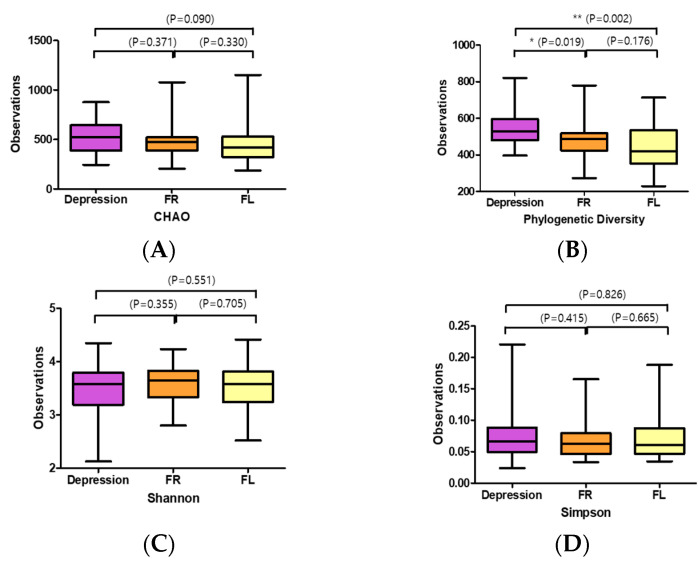
Alpha-diversity index of the gut microbiota in the subjects with depression symptoms (depression) and flavonoid-rich orange juice (FR) or equicaloric flavonoid-low orange cordial (FL) treatment groups: (**A**) Chao 1, (**B**) phylogenic diversity, (**C**) Shannon, and (**D**) Simpson. Box plots depict greater gut microbial diversity in the flavonoid-rich orange juice (FR) group than in the depression symptoms group (Depression; the participants of baseline before intervention) or flavonoid-low orange cordial (FL), according to the Shannon and Simpson indexes. The horizontal lines in the box plots represent median values; upper (Q3) and lower (Q1) ranges of the box represent the 75% and 25% quartiles, respectively.

Phylogenetic alpha-diversity indexes (Chao, Phylogenetic Diversity, Shannon, and Simpson) were used to assess gut microbial diversity in participants with depression before and after flavonoid treatment (Figure 1). Gut microbial diversity, as estimated by Chao, was greater in the Depression symptoms group (the participants of baseline before intervention) than in the FR (*p* = 0.371) and FL (*p* = 0.090) groups (Figure 1A). Moreover, the depression group plot had significantly higher phylogenetic diversity than the FR (*p* = 0.019) and FL (*p* = 0.002) group plots (Figure 1B). However, diversity as measured using the Shannon and Simpson indexes had no significant differences between the depression symptoms and flavonoid treatment groups (Figure 1C,D).

### 3.6. Changes in Microbiota Taxonomic Composition in the Before FR and FR Groups

**Figure 2:**
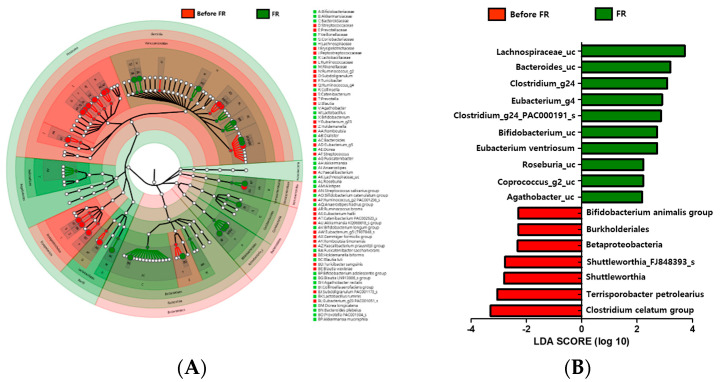
Genus level distribution and linear discriminant analysis (LDA) effect size (LEfSe) analysis revealed differences in the gut microbiota between the before FR and FR groups. (**A**) A cladogram of taxonomic differences between the before FR (red) and FR (green) groups. (**B**) Significant bacterial differences were found in the before FR (red) and FR (green) groups. The significant threshold of the LDA score is >2; flavonoid-rich orange juice treatment taxonomic compositions are indicated with a positive LDA (green) in the FR group and enriched taxonomic compositions are indicated with a negative LDA score (red) in the before FR group.

Comparisons based on taxonomy were performed to determine the differences between the microbiota of 20 individuals with depression before FR treatment group and after FR treatment group. At the phylum level, Firmicutes were more abundant in the gut in the before FR group than in the FR group. Compared with the before FR group, the Bacteroidetes and Actinobacteria phyla were increasingly abundant in the FR group. There was higher abundance of the Ruminococcaceae and Erysipelotrichaceae families in the before FR group, while the Lachnospiraceae, Bifidobacteriaceae, and Akkermansiaceae families were more abundant in the FR group. Several differences between the before FR and FR groups were observed at the genus level. *Lactobacillus, Alistipes, Roseburia*, unclassified Lachnospiraceae *(Lachnospiraceae_uc), Akkermansia, Bifidobacterium*, and *Collinsella* abundance rates were higher, while *Faecalibacterium*, *Streptococcus*, and *Eubacterium_g23* abundance rates were lower in the FR group (Figure 2A). Further, we also used metagenome analysis to validate the linear discriminant analysis (LDA) effect size (LEfSe) and found that eight families (Bifidobacteriaceae, Akkermansiaceae, Bacteroidaceae, Veillonellaceae, Coriobacteriaceae, Lachnospiraceae, Lactobacillaceae, and Rikenellaceae) were abundant in the FR group (Figure 2A). An LDA analysis (*p* < 0.05, LDA score > 2) showed that 10 taxons were more abundant in the FR group (Figure 2B). Lachnospiraceae (*Roseburia*, *Coprococcus_g2*, *Agathobacter*, *Clostridium_g24*, and *Eubacterium_g4*), Bifidobacteriaceae (*Bifidobacterium*), and Bacteroidaceae (*Bacteroides*), which were enriched in the FR group, were the major phylotypes that contributed to the difference in the gut microbiota composition between the before FR and FR groups.

### 3.7. Changes in Microbiota Taxonomic Composition in the Before FL and FL Groups

**Figure 3:**
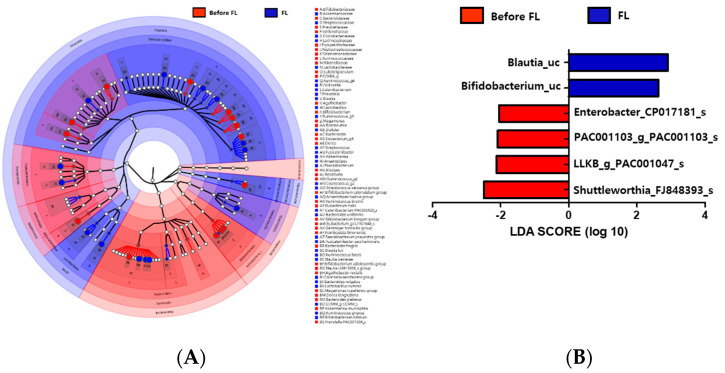
Genus level distribution and LEfSe analysis revealed differences in the gut microbiota between the before FL and FL groups. (**A**) A cladogram of taxonomic differences between the before FL (red) and flavonoid-low orange cordial (FL; blue) groups. (**B**) Significant bacterial differences were observed in the before FL (red) and FL (blue) groups. The significant threshold of the LDA score is >2; taxonomic compositions in the FL group are indicated with a positive LDA (blue), and enriched taxonomic compositions in the before FL group are indicated using a negative LDA score (red).

The gut microbiota composition of 20 individuals with depression before FL treatment group and after FL treatment groups showed differences in two phyla, Firmicutes and Bacteroidetes. The relative abundance of Firmicutes was greater in the FL group than in the before FL group, while Bacteroidetes had a lower abundance rate in this group (Figure 3A). At the family level, Ruminococcaceae, Bacteroidaceae, and Bifidobacteriaceae were less abundant in the FL group, but Lachnospiraceae, Coriobacteriaceae, Streptococcaceae, and Lactobacillaceae were more abundant in this group. *Bacteroides* and *Bifidobacterium* were enriched in the before FL group compared with that of the FL group at the genus level (Figure 3A). The LEfSe analysis also showed that the *Bacteroidetes* phylum and *Bacteroides* and *Subdoligranulum* genera were enriched in the before FL group, while the *Firmicutes* phylum and *Blautia, Streptococcus*, and *Coprococcus* genera were abundant in the FL group (Figure 3A). LDA analysis (*p* < 0.05, LDA score > 2) revealed that Lachnospiraceae (*Blautia_uc* and *Bifidobacterium_uc*) were more abundant in the FL group than in the before FL group (Figure 3B).

### 3.8. Association between Gut Microbiota and Depression

**Figure 4:**
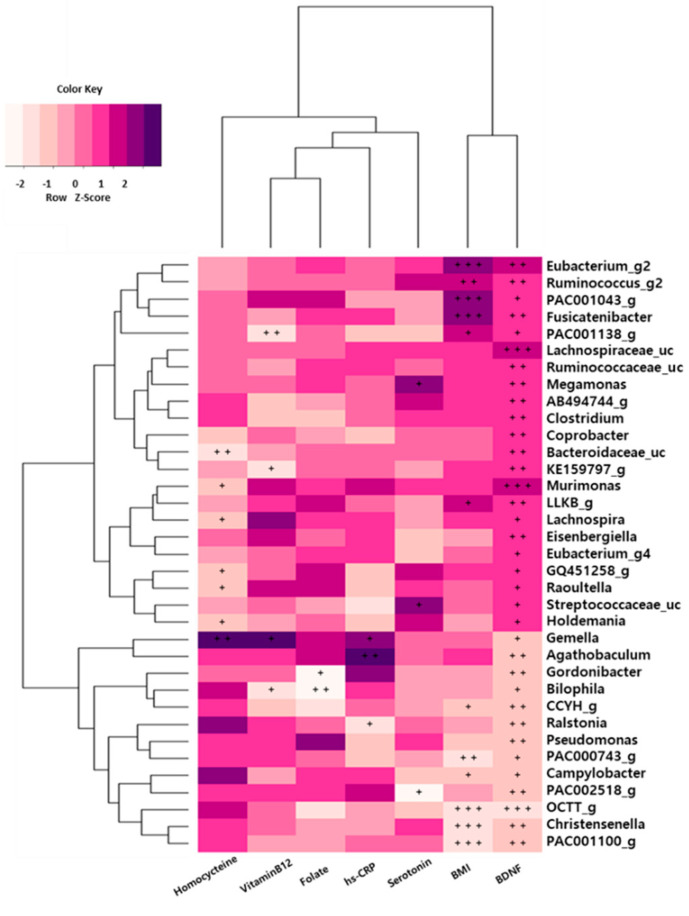
Associations of the gut microbiota with brain-derived neurotrophic factor (BDNF) levels and clinical parameters in depression symptoms group. The heat map of Spearman’s rank correlation coefficients obtained by comparing clinical parameters (BDNF scores, *p* < 0.05) and relative abundances of gut microbiota in depression symptoms group. The heat map was plotted using R software. ^+^ *p* < 0.10; ^++^ *p* < 0.05; ^+++^ *p* < 0.01.

We also evaluated the associations of serum biomarkers with the relative abundances of gut microbiota. We found that depression and serum biomarkers were closely associated with gut microbiota in the depression group (Figure 4). The serum BDNF level was significantly positively correlated with the abundance of the Lachnospiraceae family (*Lachnospiraceae_uc* and *Murimonas*) and was negatively correlated with OCTT_g (Ruminococcaceae) abundance in those with depression. The relative abundances of *Fusicatenibacter*, PAC001043_g, and *Eubacterium_g20* were positively correlated with body mass index (BMI), while those of the Ruminococcaceae family (PAC001100_g and OCTT_g) and *Christensenella* genus were negatively correlated with BMI (*p* < 0.001). Additionally, homocysteine levels were positively correlated with the abundance of the *Gemella* genus (*p* < 0.01) in the depression symptoms group.

### 3.9. FR Increased the Relative Abundance of Lachnospiraceae_uc and Bifidobacterium_uc in Depression Symptoms Group

**Figure 5:**
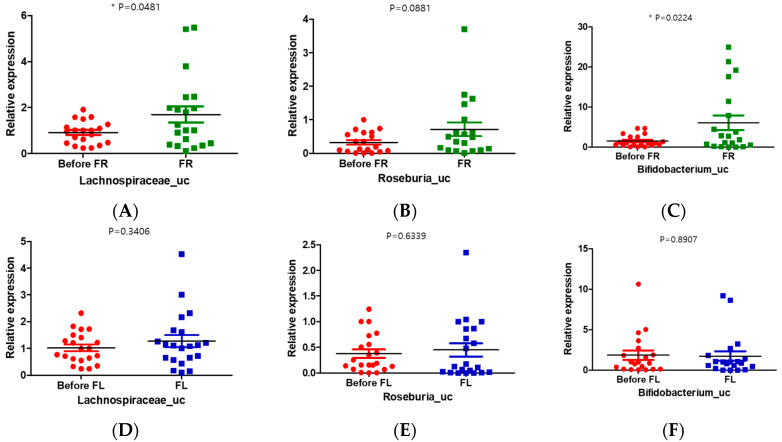
Quantitative changes in the gut microbiome in the before and after treatment (FR or FL) group. (**A**,**D**) Unclassified Lachnospiraceae (*Lachnospiraceae_uc*); (**B**,**E**) unclassified Roseburia (*Roseburia_uc*); (**C**,**F**) unclassified Bifidobacterium (*Bifidobacterium_uc*) in the FR (green) or FL (blue) groups. * *p* < 0.05.

To explore the contribution of the FR to the observed changes in gut microbial compositions in those with depression, we conducted a real-time polymerase chain reaction (PCR) experiment with stool samples from the depression symptoms group and FR and FL groups. We used the universal bacterial gene for copy control and another normal stool sample (cohort) for internal control (Table S5). The results of real-time PCR showed that the relative abundance of *Lachnospiraceae_uc* and *Bifidobacterium_uc* was significantly increased in the FR group compared to that in the before FR group (Figure 5A,C). However, the relative expression of *Lachnospiraceae_uc* was increased and that of *Bifidobacterium_uc* was decreased in the FL group compared with those in the before FL group, but there was no significant difference (Figure 5D,F). The relative expression of *Roseburia_uc* in the FR group was increased compared with that in the FL group, but no significant difference was noted (Figure 5B,E).

## 4. Discussion

This intervention study characterized the gut microbiota in the depression symptoms (CES-D scores ≥ 21) group compared with those after the flavonoid-rich (600 ± 5.4 mg/day flavonoids) or flavonoid-low (108 ± 2.6 mg/day flavonoids) treatment groups [48]. Furthermore, the associations between gut microbiota and depression were investigated, and the 8-week flavonoid intervention changed the gut microbiota taxonomic composition and diversity in the depression group. Nutrient intake was not different between the two groups, and the participants maintained work-out as usual for 8 weeks. After the 8-week intervention, we found no significant differences between FR and FL groups in the CES-D scores and the serum levels of the BDNF. However, the BDNF levels in the FR group were higher than those in the FL group in blood tests. In addition, the *p*-values of the CES-D scores in the FR group decreased significantly (*p* < 0.0001) compared to that of FL groups (*p* < 0.001) after the intervention (Table 1). The CES-D is a widely used clinical testing tool for the presence of major depression and is known to be a good choice when sampling young adults with high levels of depressive symptoms [49,50].

The flavonoids are one of the best absorbents, and some metabolites are effective to traverse the blood–brain barrier (BBB) [51]. Rodent research indicated that the citrus flavonoids hesperetin and naringenin and their relevant metabolites had been shown in the brain after oral ingestion [52]. Moreover, treatment with hesperidin may have neuroprotection, attenuated oxidative damage, and restored antioxidant enzyme activities in the frontal cortex and hippocampus [53].

In this study, the abundances of Lachnospiraceae (*Lachnospiraceae_uc* (*p* < 0.0056) and *Murimonas* (*p* < 0.0061)) were positively correlated with BDNF levels in the depression group (before flavonoid treatment), and the abundances of OCTT_g (Ruminococcaceae, *p* < 0.0002) were negatively correlated with serum BDNF levels. After groups were treated with flavonoid-rich orange juice, we observed significantly increased expression of *Lachnospiraceae_uc* in the FR group compared with that in the before FR group, as shown in real-time PCR (Figure 2B). This was in accordance with increased BDNF levels in FR treatment (Table 1). BDNF is a neurotrophin that performs multiple functions in the central nervous system and participates in the therapeutic mechanisms of antidepressants [54]. BDNF levels were significantly lower in patients with major depressive disorder (MDD) than the control group, and recovery from depression after antidepressant treatment was associated with normal serum levels of BDNF in patients with MDD [55].

Similarly, there were more changes in the taxonomic composition of the microbiota in the FR group than in the FL group. In the FR group, 10 taxons, including Lachnospiraceae, Bacteroides, and Bifidobacterium, were more abundant, but only 1 taxon (*Blautia_uc*) increased in abundance in the FL group compared to that in the before FL group. Moreover, the taxonomic abundance of Bifidobacterium was decreased in the FL group (Figure 5F and Figure S2B). Furthermore, the relative abundance of Clostridium decreased further in the FR group (Figures S1F and S2F). The prevalence of Clostridium was negatively associated with serum BDNF levels [56].

In our study, we found that the unidentified genus of Lachnospiraceae, Bacteroides, and Bifidobacterium was correlated with FR treatment, and that *Blautia_uc* (Lachnospiraceae) was correlated with FL treatment. Therefore, we found that Lachnospiraceae responds to both flavonoid-rich and low treatment in young adults with depression. Naseribafrouei et al. (2014) reported that the abundance of higher-order Bacteroidales and the Oscillibacter and Alistipes genera and lower abundance of the family Lachnospiraceae were associated with depression in the comparison of the gut microbiota between 37 patients with depression and 18 healthy controls [57]. Moreover, Jiang et al. (2015) reported that the abundance of Lachnospiraceae and Ruminococcaceae decreased in patients with MDD compared with that in the control group [58]. In our study, after the intervention, the relative taxonomic abundance of Lachnospiraceae increased in both groups (FR and FL) compared with that in the depression symptoms group. Furthermore, the abundance of genus Lachnospiraceae significantly increased (*p* < 0.0001) in the FR group compared with that in the FL group (*p* = 0.0002) (Figures S1A and S2A). Moreover, significantly increased expression of *Lachnospiraceae_uc* and *Roseburia_uc* were observed in the FR group compared with that in the FL group, as shown by real-time PCR results of stool samples (Figure 5).

In addition, the relative taxonomic abundance of Bifidobacterium, Roseburia, Ruminococcus, and Akkermansia in the FR group significantly increased compared to that in the FL group (Figures S1 and S2). The relative expression of *Bifidobacterium_uc* in the FR group was also significantly increased compared to that in the FL group (Figure 5C,F). Bifidobacterium is a genus that influences intestinal function in infants who have received healthy breast milk, while low but relatively stable Bifidobacterium counts are observed in adulthood [58]. The abundance of Bifidobacterium increases in the late stages of pregnancy in both women and mice, indicating the causative role of progesterone [59]. *Bifidobacterium bifidum* colonization increases interleukin 6 (IL-6) and IL-8 cytokine levels through NF-κB activation in mice [60]. Supplementation of Bifidobacterium has also been shown to increase the fecal levels of immunoglobulin A in young women [61] and to lead to changes in the levels of human immune cells [62]. Furthermore, exogenous probiotic microbes, such as Bifidobacterium and Lactobacillus, have been shown to reduce anxiety in human participants [63].

Relative abundances of bacterial genera, including Akkermansia spp., were significantly reduced in socially defeated animals, which was positively correlated with both anxiety and depression [64]. However, the administration of prebiotics increased the relative abundance of Akkermansia during exposure to stressors [65,66]. Moreover, polyphenol-rich cranberry extract prevented several detrimental features of the metabolic syndrome in association with the abundance of Akkermansia in the gut microbiota [67]. The abundance of Akkermansia has been suggested as a biomarker for healthy intestines and has an inverse correlation with several intestinal disorders [68]. Generally, Akkermansia and Bifidobacterium are well-known health-associated genera that protect against inflammation, promote immunomodulation, and promote healthy metabolic homeostasis [69]. These findings corroborate our study on flavonoid intervention, and the relative abundance of Akkermansia significantly increased in the FR group compared to that in the FL group.

Greater microbial diversity was found in the depression group than that in the FR and FL groups. To date, four studies on MDD have investigated and performed a microbial diversity analysis. While three studies reported no significant differences in microbial diversity [57,70,71], Jiang et al. (2015) reported greater microbial diversity in patients with MDD than in healthy individuals [56]. High microbial diversity could be easily affected by age, eating habits, and other factors [38]. Although greater diversity of bacteria is potentially beneficial to human health, the precise consequences of increased bacterial diversity for depression are still unclear [56].

Members of the Lachnospiraceae family, such as the *Roseburia*, *Blautia*, and *Coprococcus* genera, are known to break down carbohydrates into short-chain fatty acids (SCFAs) [72]. Reduction in these fermentation-related bacteria precipitates a decline in SCFA production, causing intestinal barrier dysfunction [72,73]. Moreover, SCFAs promote the differentiation of T cells and can function as a histone deacetylase inhibitor [74]; therefore, SCFAs can act as a regulator of immune homeostasis. Additionally, SCFAs are involved in neurotransmitter production [75] and neuroprotection and can penetrate the blood–brain barrier [76]; therefore, they have been proposed as potential novel antidepressants [77]. The Ruminococcaceae family was highly abundant in healthy controls compared to that in patients with MDD [56]. Moreover, the Ruminococcaceae family is characterized by anti-inflammatory activity and associated with a chronic low-grade inflammatory response [68,78]. Therefore, the relative abundance of these genera in the gut microbiome mediated the low degree of inflammation and higher intestinal barrier function. Inflammation is associated with major depression [79]. Furthermore, flavonoids exhibit a neuroprotective effect by falsifying inflammatory reactions and have potential therapeutic effects in terms of neuroprotection [80].

In this study, the use of antibiotics, probiotics, and prebiotics was not allowed for assessing the microbial community. Additionally, we served fresh, 100% pure orange juice daily as the FR, and investigated the associations between the relative abundance of the gut microbiome and depression in young adults. Although the particular association between flavonoids and depression is still unclear, we found that a high intake of flavonoids changed the relative abundance of the gut microbiome, especially the butyrate-producing Lachnospiraceae family. Therefore, we suggest the efficacy of FR than FL in improving depression.

Gut microbiota can be affected by several variables. In this study, some limitations were present, and the use of antibiotics and probiotics was not permitted. Only CES-D was used to screen depression symptoms, and the intake of all nutrients decreased in the FR group compared to those of the FL group except carbohydrate. The number of participants who had depression symptoms was small. Well-designed extensive studies with depressive patients are needed to confirm our findings. Nonetheless, this study is meaningful as it is the first study to a potential improvement effect of alterations in the microbiome due to flavonoid treatment in young adults with depressive symptoms.

## Figures and Tables

**Table 1:** Anthropometric data and blood test results at baseline and 8 weeks after intervention.

Variables	Flavonoid-Rich Orange Juice (FR, *n* = 20)		*p*-Value ^†^	Flavonoid-Low Orange Cordial (FL, *n* = 20)		*p*-Value ^†^	Δ Group Comparison ^¥^
	Baseline	After Intervention		Baseline	After Intervention		
	Mean ± SE			Mean ± SE			
Age	22.20 ± 2.608			21.45 ± 2.259			0.337 ^†^
Male	*n* = 8 (40%)			*n* = 8 (40%)			1.000
Weight, kg	66.28 ± 3.41	66.57 ± 3.40	0.382 ^†^	60.22 ± 2.32	59.98 ± 2.31	0.510 ^†^	0.672
BMI, kg/m^2^	23.45 ± 0.87	23.62 ± 0.88	0.178 ^†^	21.74 ± 0.66	21.62 ± 0.63	0.387 ^†^	0.122
Percent body fat, %	27.72 ± 1.76	27.90 ± 1.85	0.609 ^†^	25.84 ± 2.08	24.73 ± 2.18	0.052 ^†^	0.050
SBP, mmHg	121.25 ± 2.98	123.05 ± 2.78	0.520 ^†^	121.20 ± 2.39	118.40 ± 3.55	0.307 ^†^	0.117
DBP, mmHg	74.80 ± 2.01	76.20 ± 1.63	0.522 ^†^	70.55 ± 2.24	72.60 ± 1.47	0.397 ^†^	0.063
BDNF	255.30 ± 40.78	322.08 ± 42.80	0.038 ^‡^	267.23 ± 45.00	287.45 ± 53.24	0.673^‡^	0.132
Serotonin, ng/mL	151.73 ± 22.76	187.66 ± 27.12	0.219 ^†^	122.62 ± 13.37	154.23 ± 20.69	0.102 ^†^	0.058
Folate, ng/mL	6.31 ± 0.69	7.47 ± 1.00	0.013 ^†^	6.39 ± 1.45	6.72 ± 3.41	0.536 ^†^	0.057
hs-CRP, mg/L	1.76 ± 0.56	0.81 ± 0.29	0.180 ^‡^	2.03 ± 0.89	0.41 ± 0.10	0.061^‡^	0.031
Vitamin B_12_, pg/mL	517.70 ± 30.57	507.75 ± 25.80	0.694 ^†^	550.45 ± 45.51	542.00 ± 38.89	0.768 ^†^	0.143
CES-D score	30.4 ± 7.97	15.15 ± 8.95	<0.0001 ^†^	28.35 ± 6.49	17.85 ± 7.36	0.001 ^†^	0.889

BMI, body mass index; ^†^ paired *t*-test; ^‡^ Wilcoxon signed rank test; ^¥^ multiple regression analysis was applied for Δ values (income, sex, and age were adjusted).

## Supplementary Materials

The following are available online at https://www.mdpi.com/2072-6643/12/6/1815/s1, Figure S1: Relative abundance of fecal microbiota between before and after flavonoid-rich FR group, Figure S2: Relative abundance of fecal microbiota between before and after flavonoid-low FL group, Table S1: Baseline characteristics of flavonoid-rich orange juice (FR) and flavonoid-low orange cordial (FL), Table S2: Nutrient intakes of 24-h recall at baseline and 8 weeks after intervention, Table S3: The content and mass spectrometry data of the seven identified flavonoids in flavonoid-rich orange juice (FR) and flavonoid-low orange cordial (FL), Table S4: Nutritional composition of the study treatments in flavonoid-rich orange juice (FR) and flavonoid-low orange cordial (FL), Table S5: Oligonucleotides used for quantitative real-time polymerase chain reaction assays.
