# Supplementary material for: Flavonoid-Rich Orange Juice Intake and Altered Gut Microbiome in Young Adults with Depressive Symptom: A Randomized Controlled Study
Source: Nutrients. 2020 Jun 18;12(6):1815. doi: 10.3390/nu12061815 (PMC7353347; doi:10.3390/nu12061815)
Supplement: Supplementary file 1 [file nutrients-12-01815-s001.pdf]

Supplementary Table 1. Baseline characteristics of flavonoid-rich orange juice (FR) and flavonoid-low orange cordial (FL).

**Variables**	**FR (n = 20)**	**FL (n = 20)**	***p*-value**
Sex, N (%)			
Male	8 (40)	8 (40)	1.000‡
Female	12 (60)	12 (60)	1.000‡
Age, mean ± SD, year	22.20 ± 2.608	21.45 ± 2.259	0.337†
Residence type, N (%)			
Living alone	3 (15)	3 (15)	0.486¥
With roommate	8 (40)	4 (20)	
With parents	7 (35)	11 (55)	
Others	2 (10)	2 (10)	
Household income, N (%)			
< 1,000,000 won per month	1 (5)	1 (5)	0.751¥
1,000,000–2,999,999 won per month	7 (35)	4 (20)	
3,000,000–4,999,999 won per month	7 (35)	7 (35)	
>5,000,000 won per month	5 (25)	8 (40)	
Regular physical activity, N (%)			
Yes	13 (65)	7 (35)	0.058‡
No	7 (35)	13 (65)	
Alcohol drinking, N (%)			
None	2 (10)	1 (5)	0.966¥
Less than once a month	4 (20)	3 (15)	
Once a month	3 (15)	3 (15)	
2–4 times a month	9 (45)	11 (55)	
2–3 times a week	2 (10)	2 (10)	
Heavy drinking frequency, N (%)			
None	7 (35)	6 (30)	0.791¥
Less than once a month	2 (10)	3 (15)	
Once a month	8 (40)	9 (45)	
Once a week	1 (5)	2 (10)	
Almost everyday	2 (10)	0	
Smoking, N (%)			
None	15 (75)	16 (80)	0.415¥
Past smoking (no current smoking)	4 (20)	2 (10)	
Occasionally	1 (5)	0	
Always	0	2 (10)	
Stress, N (%)			
Too much	3 (15)	2 (10)	0.587¥
Pretty much	13 (65)	10 (50)	
Little	4 (20)	7 (35)	
Barely	0	1 (5)	

† Student t-test, ‡ Chi-square test, ¥ Fisher's exact test

Supplementary Table 2. Nutrient intakes of 24-h recall at baseline and 8 weeks after intervention.

Variables	Flavonoid-rich orange juice (FR, n=20)		*p*-value†	Flavonoid-low orange cordial (FL, n=20)		*p*-value†	Δ group comparison¥
	Baseline	After intervention		Baseline	After intervention		
	Mean ± SD			Mean ± SD			
Energy, kcal	1699.86 ± 764.42	1484.21 ± 486.81	0.045†	1671.86 ± 487.26	1604.72 ± 481.66	0.620†	0.379
Carbohydrate, g	216.62 ± 83.27	223.19 ± 74.92	0.675†	226.11 ± 91.71	236.76 ± 80.83	0.627†	0.879
Protein, g	67.79 ± 39.77	49.12 ± 19.32	0.014†	59.05 ± 20.14	52.02 ± 18.70	0.223†	0.197
Fat, g	61.40 ± 39.46	44.84 ± 28.34	0.039†	58.61 ± 21.57	49.23 ± 20.41	0.181†	0.480
Fiber, g	13.31 ± 7.11	17.13 ± 5.00	0.030†	18.16 ± 10.53	13.04 ± 6.33	0.061†	0.006
Calcium, mg	342.45 ± 239.52	373.47 ± 169.82	0.204‡	424.30 ± 210.12	362.65 ± 234.77	0.357†	0.246
Phosphate, mg	867.38 ± 481.07	681.52 ± 224.68	0.035†	897.49 ± 391.43	738.15 ± 254.39	0.099†	0.831
Iron, mg	12.06 ± 9.45	11.19 ± 5.98	0.433‡	11.58 ± 4.95	11.37 ± 4.12	0.885†	0.798
Sodium, mg	2802.07 ± 866.72	2180.83 ± 1161.21	0.008†	3238.51 ± 1579.80	2948.47 ± 1569.63	0.486†	0.477
Potassium, mg	1752.06 ± 998.85	1724.14 ± 664.32	0.877†	1880.32 ± 780.50	1849.98 ± 730.84	0.901†	0.994
Vitamin A, μg RE	346.30 ± 204.60	381.28 ± 161.95	0.375†	571.20 ± 647.33	439.43 ± 273.41	0.841‡	0.328
β-carotene, μg	1438.63 ± 1001.52	1512.79 ± 1042.29	0.794†	2503.34 ± 4090.43	1946.34 ± 1632.73	0.898‡	0.550
Thiamin, mg	1.63 ± 1.07	1.54 ± 0.57	0.715†	1.59 ± 0.88	1.41 ± 0.51	0.378†	0.755
Riboflavin, mg	1.16 ± 0.67	1.15 ± 0.55	0.957†	1.49 ± 0.71	1.06 ± 0.43	0.010†	0.083
Niacin, mg	10.60 ± 6.31	10.16 ± 5.16	0.852‡	11.28 ± 4.02	8.87 ± 3.26	0.044†	0.255
Pyridoxine, mg	1.42 ± 0.96	1.17 ± 0.49	0.765‡	1.16 ± 0.37	1.20 ± 0.42	0.696†	0.189
Folate, μg	295.90 ± 206.56	392.77 ± 125.09	0.000†	351.87 ± 183.67	325.39 ± 159.24	0.512†	0.046
Vitamin B_{12}, μg	4.62 ± 4.84	3.86 ± 3.46	0.002‡	4.73 ± 3.88	5.08 ± 5.44	0.779†	0.476
Vitamin C, mg	56.85 ± 74.51	146.41 ± 19.55	0.002‡	42.56 ± 30.29	102.46 ± 30.80	<0.0001†	0.133

†Paired t-test, ‡Wilcoxon signed rank test, ¥Student t-test

Supplementary Table 3. The content and mass spectrometry data of the seven identified flavonoids in flavonoid-rich orange juice (FR) and flavonoid-low orange cordial (FL).

**Aglycones**	**Peak No.**	**Individual flavonoids**	**MW**	**Fragment ions (m/z)**	**FR**	**FR**
					**(mg/100 g DW)**	
Naringenin (m/z 273)	4	naringenin 7-O-rutinoside (narirutin)	580	603, 581, 435, 419, 273	28.3 ± 0.3	3.6 ± 0.1
	3	naringenin 7-O-rutinoside-4'-O-glucoside (narirutin 4'-O-glucoside)	742	765, 743, 581, 435, 273	10.4 ± 0.1	1.1 ± 0.0
Isosakuranetin (m/z 287)	7	isosakuranetin 7-O-rutinoside (didymin)	594	617, 595, 449, 433, 287	5.4 ± 0.3	1.0 ± 0.0
Hesperetin (m/z 303)	6	hesperetin 7-O-rutinoside (hesperidin)	610	633, 611, 465, 449, 303	74.2 ± 0.5	21.1 ± 0.6
Apigenin (m/z 271)	2	apigenin 6,8-di-C-glucoside(vicenin-2)	594	617, 595, 577, 559, 541, 475, 457, 439, 409	34.8 ± 0.2	1.4 ± 0.0
Luteolin (m/z 287)	1	luteolin 6,8-di-C-glucoside(lucenin-2)	610	633, 611, 593, 575, 557, 491, 473, 455, 425	2.3 ± 0.0	0.1 ± 0.0
Isorhamnetin (m/z 317)	5	isorhamnetin 3-O-rutinoside (narcissin)	624	647, 479, 317	2.5 ± 0.3	0.1 ± 0.0
		Total flavonoids			157.9 ± 1.4	28.4 ± 0.7

All samples were analyzed in the positive ion mode (*m/z*, $[M+H]^+$) using UPLC-DAD-QToF/MS

Each value calculated as means ± SD of three replicates using an internal standard (galangin 20 ppm)

Supplementary Table 4. Nutritional composition of the study treatments in flavonoid-rich orange juice (FR) and flavonoid-low orange cordial (FL).

**Nutrients per 190 g (1 serve)**	**Treatment**	
	**FR**	**FL**
	(% Daily Value)	
Energy, kcal	68	60
Total Carbohydrate, g	16 (6)	15 (15)
Sugar, g	14	15
Sodium, mg	0 (0)	25 (1)
Total fat, g	0 (0)	0 (0)
Saturated fat, g	0 (0)	0 (0)
Trans fat, g	0 (0)	0 (0)
Cholesterol, mg	0 (0)	0 (0)
Protein, g	0 (0)	0 (0)

Supplementary Table 5**.** Oligonucleotides used for quantitative real-time polymerase chain reaction assays.

Species	Primer sequences		Size	Reference
	**Forward**	**Reverse**		
Lachnospiraceae_uc	AACAGAGGAGACAGGTGGTG	GCTTCCCTTTGTTTACGCCA	228	This study
Roseburia_uc	CGCAGCAAACGCAATAAGTA	ACCACTGCTCCGAAGAGAAA	205	This study
Bifidobacterium_uc	TCGGGGTGAGTGTACCTTTC	AATTCCAGTCTCCCCTACCG	184	This study
Universal primers	CGGTGAATACGTTCYCGG	AAGGAGGTGATCCRGCCGCA	172	Suzuki et al. 2000

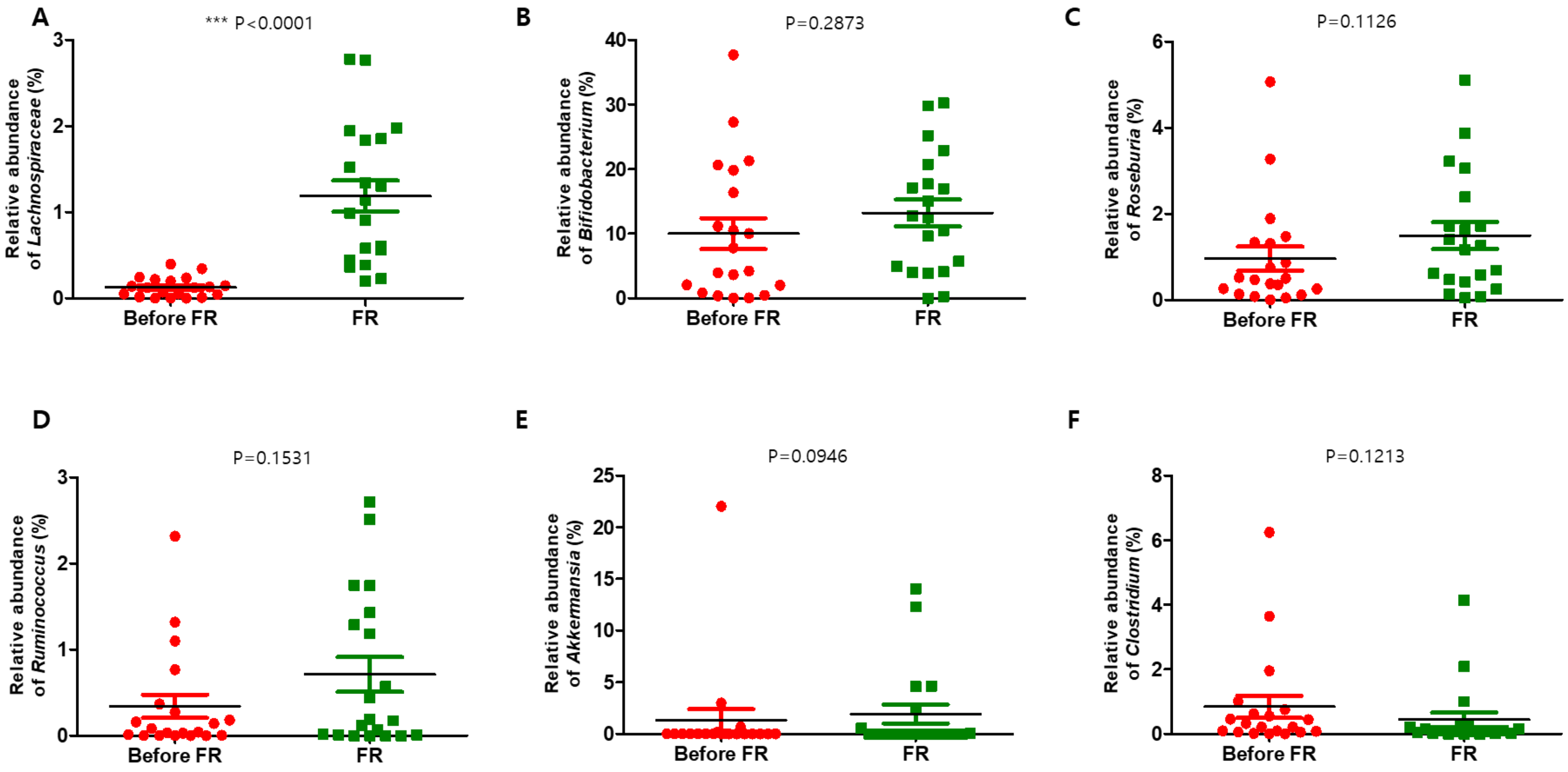

Supplement figure 1. Relative abundance of fecal microbiota between before and after flavonoid-rich FR group.

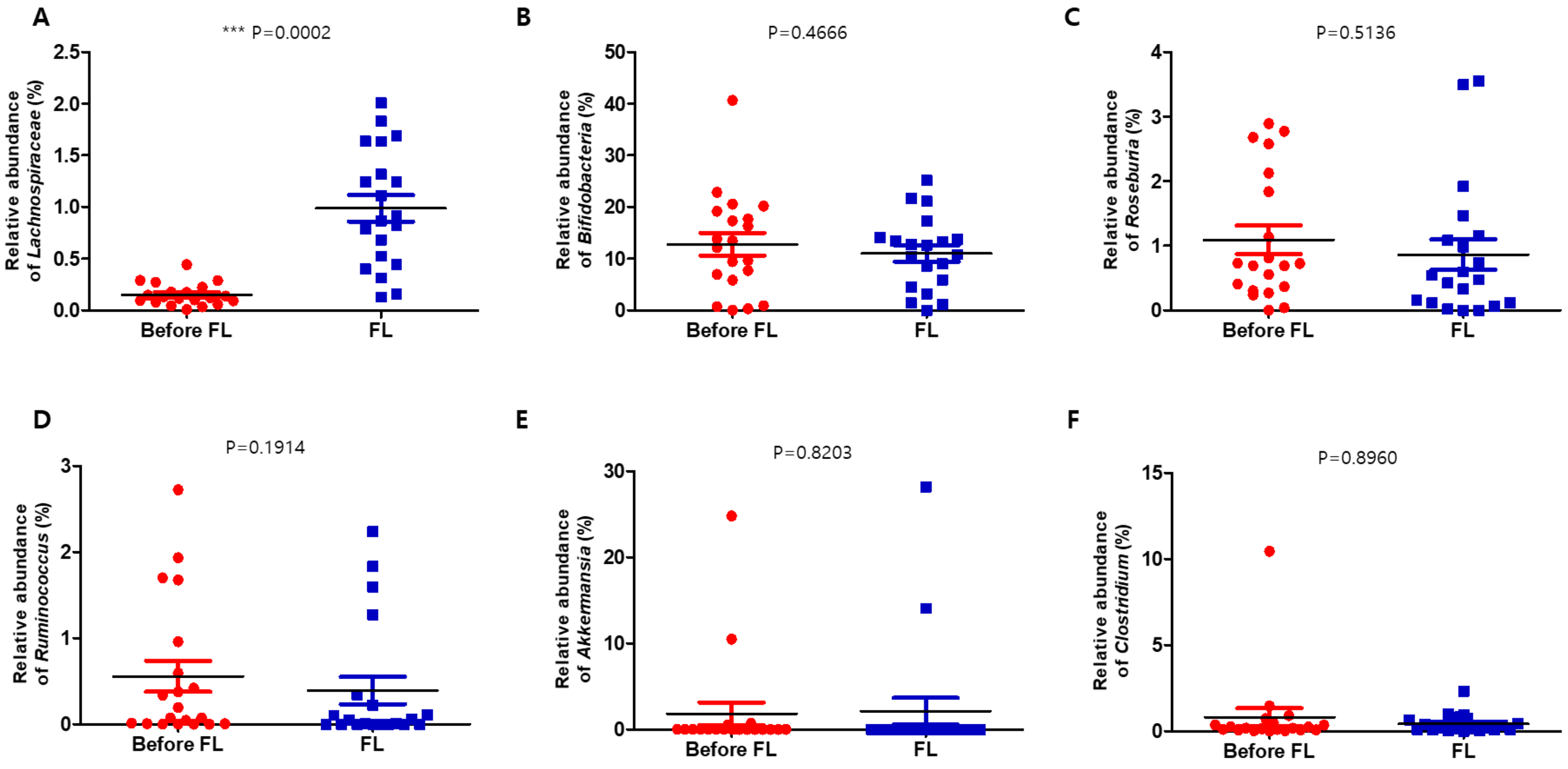

Supplement figure 2. Relative abundance of fecal microbiota between before and after flavonoid-low FL group.
